# Editorial: The Warburg Effect Regulation Under Siege: the Intertwined Pathways in Health and Disease

**DOI:** 10.3389/fcell.2019.00080

**Published:** 2019-05-15

**Authors:** Concetta Bubici, Salvatore Papa

**Affiliations:** ^1^Division of Biosciences Institute of Environment, Health and Societies, Department of Life Sciences, College of Health and Life Sciences, Brunel University London, Uxbridge, United Kingdom; ^2^Cell Signaling and Cancer Laboratory, Leeds Institute of Cancer and Pathology, Faculty of Medicine and Health, University of Leeds, St. James' University Hospital, Leeds, United Kingdom

**Keywords:** angiogenesis, aerobic glycolysis, cancer biology, inflammation, immunity

In the 1920s, the biochemist Otto Warburg observed that, unlike normal cells, cancer cells catabolize glucose into lactate under aerobic conditions (hence the name “The Warburg Effect” or aerobic glycolysis) (Warburg et al., 1927). For eight decades, the Warburg's observation was almost ignored, as only limited evidence indicated that aerobic glycolysis is characteristic of nearly all types of cancer. Over the last decade, with the development of modern analytical tools, we have witnessed a resurgence of interest in the study of the Warburg effect and the mechanistic basis of its occurrence in cancer. Not surprising, Hanahan and Weinberg (2011) in their revised list of “Hallmarks of Cancer” have included the Warburg effect as an emerging hallmark of cancer. More recently, however, a plethora of detailed investigations have also revealed crucial roles for the Warburg effect in a number of homeostatic processes, including high cell turnover and proliferation, immune responses, and brain development. To highlight the importance of the Warburg effect in health and disease, we are delighted to introduce our Research Topic containing a diversity of Review and Research Articles that will discuss the ground-breaking discoveries and advances in the field.

The Research Topic covers progress in understanding the role of the Warburg effect in cancer, immunity, inflammation, atherosclerosis, angiogenesis, and tissue homeostasis. The collection of articles in this Research Topic represents a valuable platform where the Warburg Effect is discussed from different perspectives, including how intrinsic and extrinsic regulatory pathways control aerobic glycolysis in normal and diseased conditions, and appropriately discusses the molecular basis for its inhibition in an array of human diseases.

The opening article of our Series is the detailed review by Wiese and Hitosugi that summarizes the complex role and regulation the key driver of Warburg effect, the pyruvate kinase M2 isoform (PKM2), in different cell types. The pyruvate kinase (PK) is a homotetrameric glycolytic enzyme that exists in mammals as four isoforms (PKL, PKR, PKM1, and PKM2) with different expression patterns. Its enzymatic activity catalyses the conversion of phosphoenolpyruvate (PEP) to pyruvate. The authors highlight the paradox that unlike other glycolytic enzymes, for which it is required an elevated expression and activity, PKM2 catalytic activity has to be maintained down to promote the Warburg effect in cancer cells. Indeed, it has been proposed that low PKM2 activity allows PEP and upstream glycolytic intermediate to accumulate and flow to anabolic pathways; when its activity is elevated the upstream glycolytic intermediate flow to form pyruvate, which is then converted to lactate or enters mitochondria for complete oxidation. Other than the allosteric regulation by glycolytic intermediates, post-translational modifications alter the protein localization, enzymatic activity, and stability of PKM2 to promote the Warburg Effect. Above all, tyrosine phosphorylation of PKM2 (i.e., Tyr105) by Tyrosine Kinases results in decreased PKM2 activity, and this decrease promotes the Warburg Effect (Hitosugi et al., 2009; Wiese and Hitosugi). On the other hand, it has been shown that threonine phosphorylation (i.e., Thr365) via c-Jun N-terminal Kinase (JNK) results in increased PKM2 activity and consequential apoptosis of cancer cells (Iansante et al., 2015; Papa et al., 2019), highlighting the necessity for highly proliferating cancer cells to preserve low PKM2 activity. The authors highlight the complex role of PKM2 with anti- and pro-tumorigenic function in cancer development depending on cancer type.

By employing population and single cell time-lapse imaging approaches, Lucantoni et al. demonstrate that the dual inhibition of glycolysis and mitochondrial respiration increase cell death and decrease clonogenic capacity of breast cancer cells. Importantly, it was shown that the treatment of breast cancer cells with the mitochondrial fission inhibitor MDIVI-1 alone increases aerobic glycolysis while had no effect on mitochondrial morphology. Suppressing the MDIVI-1-mediated increased glycolysis with 2-deoxy-D-glucose (2-DG)—a derivative of glucose that serves as a glycolytic inhibitor—resulted in a synthetical lethal effect in breast cancer cells.

In another research article focusing on liver cancer, Lee et al. show that glycolytic enzymes involved in glycolysis and mitochondrial oxidative metabolism (OXPHOS) are highly expressed in livers of patients with hepatocellular carcinoma (HCC) compared to healthy livers, suggesting that glycolysis cooperates with OXPHOS to sustain fast cellular proliferation in HCC. The authors also show that, like HCC, aerobic glycolysis is increased in livers of patients with cirrhosis, a chronic inflammatory liver condition predisposing to the development of HCC. However, in contrast to a general increase in expression levels of genes involved in glycolysis, OXPHOS genes remained at the same level to healthy livers in cirrhotic livers. This suggests aerobic glycolysis may represent a marker for early detection and chemoprevention of HCC. The authors also highlight the fact that aerobic glycolysis is not only a distinctive phenotype of tumors but is involved in pre-malignant conditions. A review article by Hou and Syn provides an in-depth view of the metabolic changes that occur during hepatic fibrogenesis, highlighting the impact of the Warburg effect in the activation of hepatic stellate cells such as the transdifferentiation of quiescent perisinusoidal liver resident cells into proliferative, contractile, and fibrogenic cells that is the core of liver fibrosis, which drives the progression of chronic liver diseases toward liver cirrhosis and hepatic failure.

Changes in glycolytic and mitochondrial metabolism have an impact on the skeletal muscle fiber composition and are reviewed by Julien et al. in this issue. The authors describe the metabolic characteristic of muscle fibers and evaluate the metabolite-dependent intracellular pathways that influence fiber composition. In particular, the authors discuss that during extensive exercise a rapid increase of glycolysis occurs in skeletal muscle fibers and pyruvate is converted to lactate, reminiscent of the Warburg effect in cancer cells. This metabolic swift is under control of specific metabolite-dependent cell signaling and transcriptional programs. A better understanding of these programs may facilitate therefore the therapy progress against aging and neurodegenerative diseases like amyotrophic lateral sclerosis, Huntington's disease, and Alzheimer's disease.

We were also thrilled to read a review article by Theodorou and Boon discussing the role of glucose, fatty acid, and amino acid metabolism in endothelial cells in response to various physiological and pathological stimuli. Their report suggests that interfering with the glycolytic metabolism in endothelial cells may provide therapeutic strategy for atherosclerosis, even in the presence of risk factors such as diabetes and obesity. In support of this idea, Fitzgerald et al. review how changes in endothelial cell metabolism affect endothelial cell fate during physiological sprouting, as well as during angiogenesis. Targeting metabolism in endothelial cells may have therapeutic potential for pathologies associated with angiogenesis such as cancer.

Metabolic pathways have also been implicated in the differentiation and activation of immune cells. Salmond reviews how the mechanistic target of rapamycin (mTOR) pathway regulates the metabolic reprogramming of specialized helper T cell (Th) subsets and discusses the role of glycolytic metabolism in T cell subsets. While the differentiation and effector functions of inflammatory Th1, Th2, and Th17 cells relies on engagement of aerobic glycolysis where mTOR signals play a key role in these processes, memory T cells and Tregs are dependent on fatty acid oxidation pathways. Although it was believed that compared to resting dendritic cells (DCs), activated DCs possess only increased glycolytic metabolism, Du et al. review recent progress in understanding the metabolic changes occurring in different DC subsets and outstanding questions in the field. The review article by Sieow et al. provides an overview on how myeloid cells undergo metabolic reprogramming in the tumor microenvironment and covers implications for cancer immunotherapies.

Following these articles on the Warburg effect, Rosenzweig et al. report a very interesting review article discussing a new perspective that goes beyond the Warburg effect. In particular, they describe how the one-carbon metabolism is regulated in cancer and normal proliferating cells. One-carbon metabolism supports multiple physiological processes that include nucleotide biosynthesis, amino acid homeostasis, methylation, and redox defense. The authors argue that one-carbon metabolism is therefore an essential integrator of the nutritional status of proliferating cells that serves as building blocks for biosynthesis, epigenetic, and redox reactions, and discuss how dysregulated oncogenic signals control these metabolic pathways to support and sustain high rates of proliferation and cell survival essential for tumor growth.

As the implications of the Warburg effect in health and disease continue to emerge, we are entering into a renaissance period for metabolism research. Since the Warburg's original observation, we have learnt a lot about the effect of signal transduction on metabolic pathways and how cells rapidly reprogram their metabolism although much remains to be discovered.

This Research Topic has attracted two original research articles and a considerable number of remarkable review articles providing a comprehensive view of the current knowledge on the molecular pathways and regulation of the Warburg effect and its implications in homeostasis and pathology and relevance in translational research.

We thank all authors and referees for their contributions in producing this special collection. Last but not least, we thank the Frontiers Editorial Office and the Journal Development Team for their dedication and work that made this Research Topic possible.

## Author Contributions

The guest editors conceived the research topic and wrote the editorial.

### Conflict of Interest Statement

The authors declare that the research was conducted in the absence of any commercial or financial relationships that could be construed as a potential conflict of interest.
